# First-line tuberculosis drug resistance patterns and associated risk factors in Germany, 2008-2017

**DOI:** 10.1371/journal.pone.0217597

**Published:** 2019-06-12

**Authors:** Saskia Glasauer, Doris Altmann, Barbara Hauer, Bonita Brodhun, Walter Haas, Nita Perumal

**Affiliations:** 1 Institute for Medical Information Processing, Biometry and Epidemiology—IBE, LMU Munich, Munich, Germany; 2 Department for Infectious Disease Epidemiology, Robert Koch Institute, Berlin, Germany; Instituto de Diagnostico y Referencia Epidemiologicos, MEXICO

## Abstract

**Background:**

Drug-resistant tuberculosis (TB), especially multidrug-resistant TB (MDR-TB), poses a threat to public health. While standard surveillance focuses on Rifampicin and/or Isoniazid resistance, little is known about other resistance patterns. This study aims to identify predominant drug resistance (DR) patterns in Germany and risk factors associated with them in order to inform diagnostic and treatment strategies.

**Methods:**

Case-based TB surveillance data notified in Germany from 2008–2017 were utilized to investigate DR and MDR-TB patterns for Isoniazid (H), Rifampicin (R), Pyrazinamide (Z), Ethambutol (E), and Streptomycin (S). Predominant patterns were further analyzed stratified by sex, age, country of birth, prior TB, and disease site. Multivariable logistic regression was conducted to determine risk factors associated with any resistance, MDR-TB, and complete HRZES resistance.

**Results:**

26,228 cases with complete DST results were included in the study, among which 3,324 cases had any DR (12.7%). Four patterns were predominant, representing about ¾ of all cases with any resistance (S: 814 [3.1%]; H: 768 [2.9%]; HS: 552 [2.1%]; Z: 412 [1.6%]). High proportions of S and H resistances were found among both German and foreign-born populations, especially those born in Eastern Europe, and were unexpectedly high among children (H: 4.3%; S: 4.6%). Foreign-born cases had significantly higher proportion of any resistance (16.0%) and MDR-TB (3.3%) compared to German-born cases (8.3% and 0.6%). Of 556 MDR-TB cases, 39.2% showed complete HRZES resistance. Logistic regression revealed having prior TB and being foreign-born as consistently strong risk factors for any DR, MDR-TB, and complete HRZES resistance.

**Conclusions:**

DR patterns observed in Germany, particularly for MDR-TB were more complex than expected, highlighting the fact that detailed drug-testing results are crucial before incorporating HRZES drugs in MDR-TB treatment. Furthermore, the relatively high rate of H-resistance in Germany provides strong rationale against the use of only H-based preventive therapy for LTBI.

## Introduction

Tuberculosis (TB) is the ninth leading cause of death worldwide and the leading cause of death from a single infectious agent, exceeding even HIV/AIDS [1]. The World Health Organization (WHO) estimates that in 2017, approximately 10 million people were diagnosed with TB and 1.6 million people died due to the disease [1]. About one quarter of the world’s population is further estimated to be latently infected [1]. As a result, TB control is a top priority on the global health agenda. One of the WHO´s End TB strategy targets aims to reduce the global incidence to <100 TB cases per million by 2035 [2]. The WHO has also developed a Framework for low-incidence countries [3], asking them to reach pre-elimination phase (<10 cases per million) by 2035 and elimination (<1 case per million) by 2050 or earlier [3]. One of the biggest obstacles in achieving TB elimination is the emergence of drug resistance [1]. In 2017, 558,000 cases were estimated to be resistant to Rifampicin (R), the most effective first-line anti-tuberculosis drug [1], with 82% suffering from multidrug-resistant TB (MDR-TB) [1], which is defined as the resistance to at least Rifampicin and Isoniazid, another potent anti-TB drug [1]. Tackling MDR-TB is an especially important cornerstone of the increasingly urgent global fight against antimicrobial resistance (AMR) [4].

A number of experts have suggested the lack of political commitment, inadequate healthcare systems, poor disease management, unsound drug policies, and a long-standing neglect in research as having facilitated the global rise in drug-resistant TB and MDR-TB [5, 6]; the increase in MDR-TB is especially concerning due to its prolonged, difficult, and potentially toxic treatment. In 2016, in the European Economic Region, almost two-thirds of MDR-TB cases were notified as having failed, defaulted, or died during treatment [7]. Inadequate MDR-TB treatment facilitates the development of extensively drug resistant TB (MDR-TB plus resistance against one Fluoroquinolone and one injectable; XDR TB) and paves the way for further transmission [5]. Thus, resistant, and especially MDR-TB, pose an immense threat to public health [8].

With almost 20% of R-resistant (RR) or MDR-TB cases in 2016, the WHO European Region has the highest regional burden of MDR-TB in the world [6, 9]. In 2016 alone, 19% of new TB cases and 55% of previously treated cases were diagnosed with RR/MDR-TB, which is considerably higher than the global average [10]. Eastern European countries present the highest resistance rates in Europe [6, 11]. Studies have further shown that MDR-TB strains have been imported into and transmitted throughout the EU/EEA region [5, 11–14].

Germany represents a low TB incidence country, with 5,486 cases in 2017 and a notified incidence of 6.7/100,000 [15]. In the past 10 years, the drug resistance trend in Germany has fluctuated slightly, peaking in 2013, but has remained overall stable at 2–3%. In 2017, drug resistance against at least one of the five standard anti-TB drugs H, R, Pyrazinamide (Z), Ethambutol (E) and Streptomycin (S) was notified in 431 (11.9%) cases with available information (information needed be available for at least H and R resistances). MDR-TB was reported in 109 cases (3.0%), of which 4 were reported as having XDR-TB [15]. Even within the context of Germany’s low TB incidence, however, it is important to remain attentive to epidemiological changes in drug-resistant TB in order to inform clinical decision-making on appropriate drug regimens [16].

As TB treatment has shifted from standardized to more individualized approaches [17–19] information on drug resistance patterns within the population has become ever more relevant and important. Furthermore, due to the high prevalence of MDR-TB among R-resistant cases, R resistance is considered as surrogate for MDR-TB and amongst the WHO-endorsed rapid drug susceptibility testing (DST) tools, one of the most frequently utilized tools tests only for R resistance [20–23]. However, this approach is questioned by some experts and, hence, additional DST for H, especially in low-incidence settings, is recommended [24, 25]. Here too, information on drug resistance patterns is crucial to inform the utilization of different resistance diagnostic methods.

To our knowledge, with the exception of one study conducted by the European Centre for Disease Prevention and Control, covering a period from 2007 to 2012, there is currently no analysis available on drug resistance patterns in low TB incidence countries in Europe, such as Germany [16]. Other studies who have addressed this question either limited their analysis to certain population groups [26–28] or were conducted using surveillance data from high TB incidence countries with very different TB epidemiology [29–31]. As a result, this study aims to analyze German national TB surveillance data for the predominant drug resistance patterns for the five standard anti-TB drugs (Isoniazid—H, Rifampicin—R, Pyrazinamide—Z, Ethambutol–E, Streptomycin–S; HRZES) and the factors associated with them, in order to inform diagnostic and treatment strategies for TB patients in low incidence countries such as Germany. The objectives of this study are to determine (i) frequency and distribution of drug resistance patterns over a 10-year period and (ii) demographic factors associated with specific drug resistance patterns.

## Methods

### Data source and collection

TB is a notifiable disease in Germany [32]. District health authorities report, via their respective state health departments case-based data electronically to the national surveillance database (SurvNet@rki, [33]) of the Robert Koch Institute (RKI). The data comprises of, among others, information on case demographics, site of disease, prior TB disease, bacteriological findings, DST results, and treatment outcome.

### Case inclusion and definitions

For study inclusion, a case had to meet the following criteria: fulfil the RKI reference case definition for active TB disease [34], be notified between the years 2008 to 2017, be culture positive, and have been tested for all five HRZES drugs and have DST results available. The cut-off date for the data extraction was March 1^st^, 2018. As S was considered and used as a first-line TB drug until 2011 covered by the study period, it was included in the analyses to reflect drug resistance dynamics in Germany. Z was also included, despite inconsistent international guidelines regarding the reliability and quality assurance of its DST [35, 36] as interlaboratory tests in Germany revealed good performance; Z has become increasingly important in newer therapy regimens [37, 38]. Information on second-line drugs could not be included in this analysis due to insufficient data completeness.

Cases <15 years were categorized as children; cases between 15 and 59 years were categorized as adults, and cases > = 60 years as elderly. Any drug resistance was defined as resistance against at least one of the HRZES drugs, any R and any H resistance was defined as resistance to at least R or H, respectively. MDR-TB was defined as being resistant against at least H and R. A case was considered a previous case, when a prior diagnosis of TB disease was notified.

### Data analysis

For the purposes of this study, the following variables were extracted from the national surveillance database: age, sex, country of birth, prior TB diagnosis (yes/no; used as surrogate for prior treatment due to a much higher level of data completeness), main site of disease (pulmonary vs. extrapulmonary), presence of MDR-TB (yes/no), individual DST results for HRZES (resistant vs. susceptible). For children <15 years of age, country of birth (born in Germany vs. born outside Germany) of the father and/or mother were also extracted.

Drug resistance patterns and their distribution were first analyzed descriptively and presented as case counts and proportions stratified by sex, age-group, country of birth, prior TB, and site of disease. Trends in HRZES monoresistances and MDR-TB, including MDR-TB with additional resistances to first line drugs, were analyzed using the Chi^2^ test for linear trends. For all other analyses, data were pooled over the entire study period. Case counts were compared using the Chi^2^ test and the corresponding p-values specified.

Further exploratory analysis was then conducted using univariable logistic regression. Finally, multivariable logistic regression analyses were performed to determine the association between age, sex, country of birth, previous TB, and site of disease (independent variables) and the presence of any resistance, MDR-TB, and resistance against all five drugs (dependent variables). The variable age was categorized into the following three groups before inclusion into the models: children (<15 years), adults (15–59 years) and elderly (60+ years). This was conducted in order to elucidate the expected differences in drug resistance patterns between the different age groups due to birth cohort effects as well as effect modification by age. Cases with missing values for any of the included variables were excluded from the analyses. A p-value of <0.01 was considered significant for the descriptive analyses, while 95% confidence intervals were calculated for the logistic regression analyses. All data were analyzed in RStudio Version 1.0.153 [39]. All data were collected in accordance with the German ‘Protection against Infection Act’ (‘Infektionsschutzgesetz’) and data protection guidelines were strictly followed. Informed consent was deemed not necessary as data were fully anonymized before analysis.

## Results

**Fig 1:**
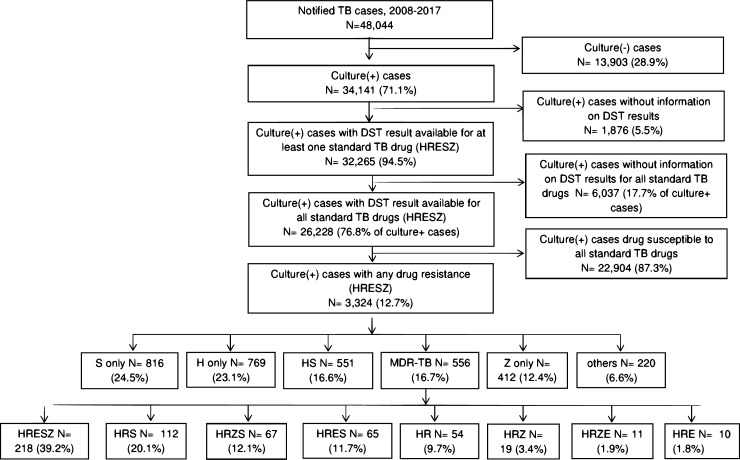
Flow chart describing case selection process and observed predominant drug resistance patterns for the five anti-TB drugs, and drug resistant patterns in MDR-TB cases, Germany 2008–2017: Isoniazid/H, Rifampicin/R, Pyrazinamide/Z, Ethambutol/E, and Streptomycin/S; DST = drug susceptibility testing.

Between 2008 and 2017, 48,044 active TB cases that met the RKI reference definition were notified in Germany. 41,932 cases (87.3%) had a culture result available, of which 34,141 were culture positive (81.4%). Of these, DST results were available for all 5 drugs (HRZES) for 26,228 cases or 76.8% (Fig 1). These 26,228 cases were considered to be the final study population.

For those with available information, foreign-born cases comprised 57.1% of all included cases. Similarly, for those with available information, the median age of the entire study population was 45 years and 63.2% were male. Median age among foreign-born cases was 35 years and 62.3% were male (German-born: 58 years and 64.7%, respectively). 12.5% of all cases with information on prior diagnosis were previously diagnosed with TB. Further case characteristics are shown in Table 1.

**Table 1:** Characteristics of study population: All culture-positive TB cases with DST results for HRZES by drug resistance patterns in Germany, 2008–2017.

Characteristics	Total number of cases		Pan-susceptible		Monoresistant		MDR TB		Others	
	N	%	N	%	N	%	N	%	N	%
**Total**	26, 228		22,904		2, 080		556		688	
**Sex**										
**(n = 26,183)**										
Female	9,627	36.8%	8,347	36.5%	810	39.1%	202	36.3%	268	39.2%
Male	16,556	63.2%	14,523	63.5%	1,263	60.9%	354	63.7%	416	60.8%
Unknown	45		34		7		0		4	
**Age groups [years]**										
**(n = 26,226)**										
0–14	588	2.2%	484	2.1%	65	3.1%	14	2.5%	25	3.6%
15–39	10,534	40.2%	8,948	39.1%	935	45.0%	341	61.3%	310	45.1%
40–59	7,173	27.4%	6,229	27.2%	549	26.4%	155	27.9%	240	34.9%
60+	7,931	30.2%	7,242	31.6%	530	25.5%	46	8.3%	113	16.4%
Unknown	2		1		1		0		0	
**Country of birth**										
**(n = 25,569)**										
Foreign	14,600	57.1%	12,263	54.9%	1,333	65.7%	478	87.2%	526	78.3%
Germany	10,969	42.9%	10,056	45.1%	697	34.3%	70	12.8%	146	21.7%
Unknown	659		585		50		8		16	
**Previous TB**										
**(n = 22,555)**										
No	19,735	87.5%	17,432	88.3%	1,529	86.4%	281	60.2%	493	84.7%
Yes	2,820	12.5%	2,304	11.7%	241	13.6%	186	39.8%	89	15.3%
Unknown	3,673		3,168		310		89		106	
**Site of disease**										
**(n = 26,195)**										
Pulmonary	21,254	81.1%	18,608	81.3%	1,591	76.7%	493	88.8%	562	81.7%
Extra-pulmonary	4,941	18.9%	4,270	18.7%	483	23.3%	62	11.2%	126	18.3%
Unknown	33		26		6		1		0	

### Trends in drug resistance over time

**Fig 2:**
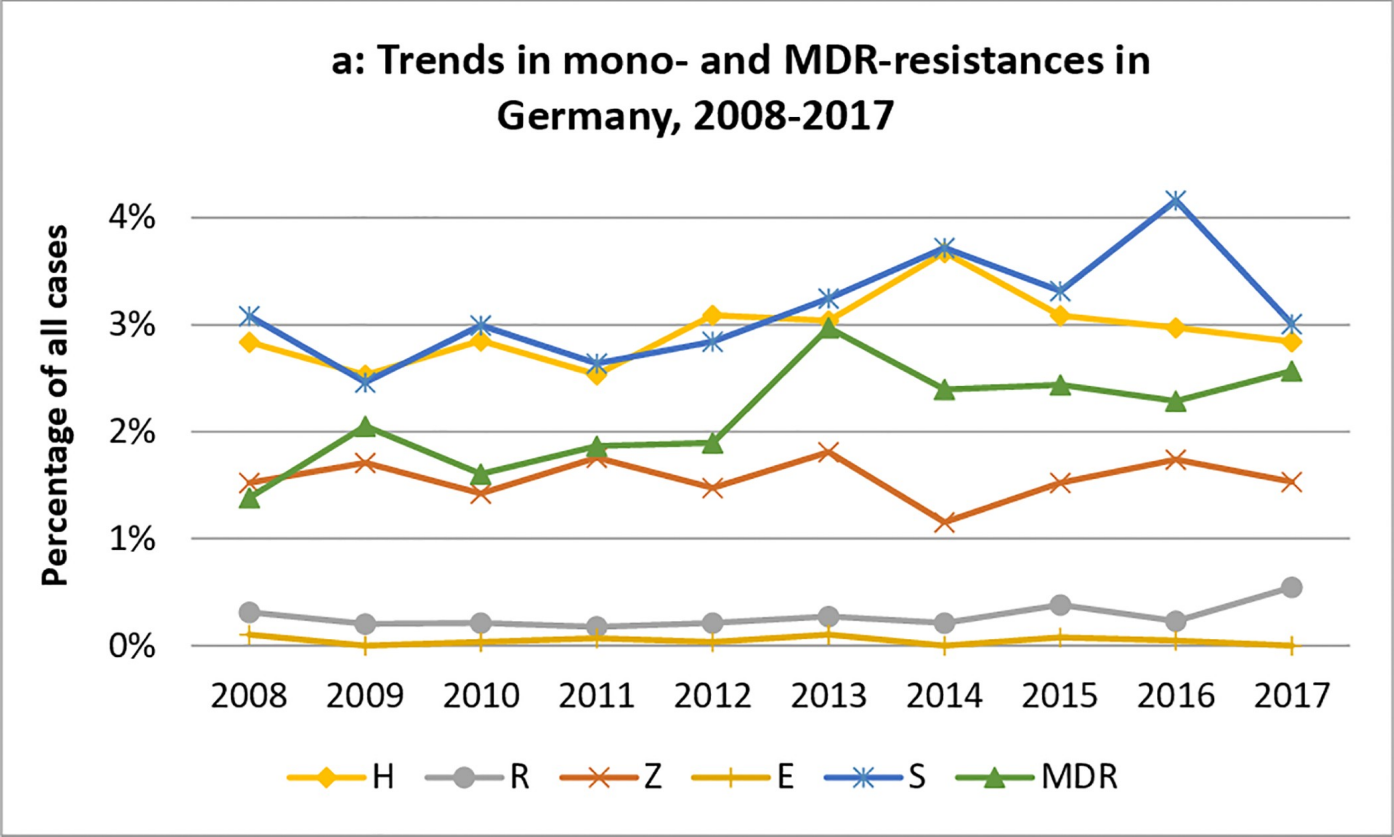
Trends in monoresistant TB and MDR-TB notifications between 2008 and 2017 in Germany.

**Fig 3:**
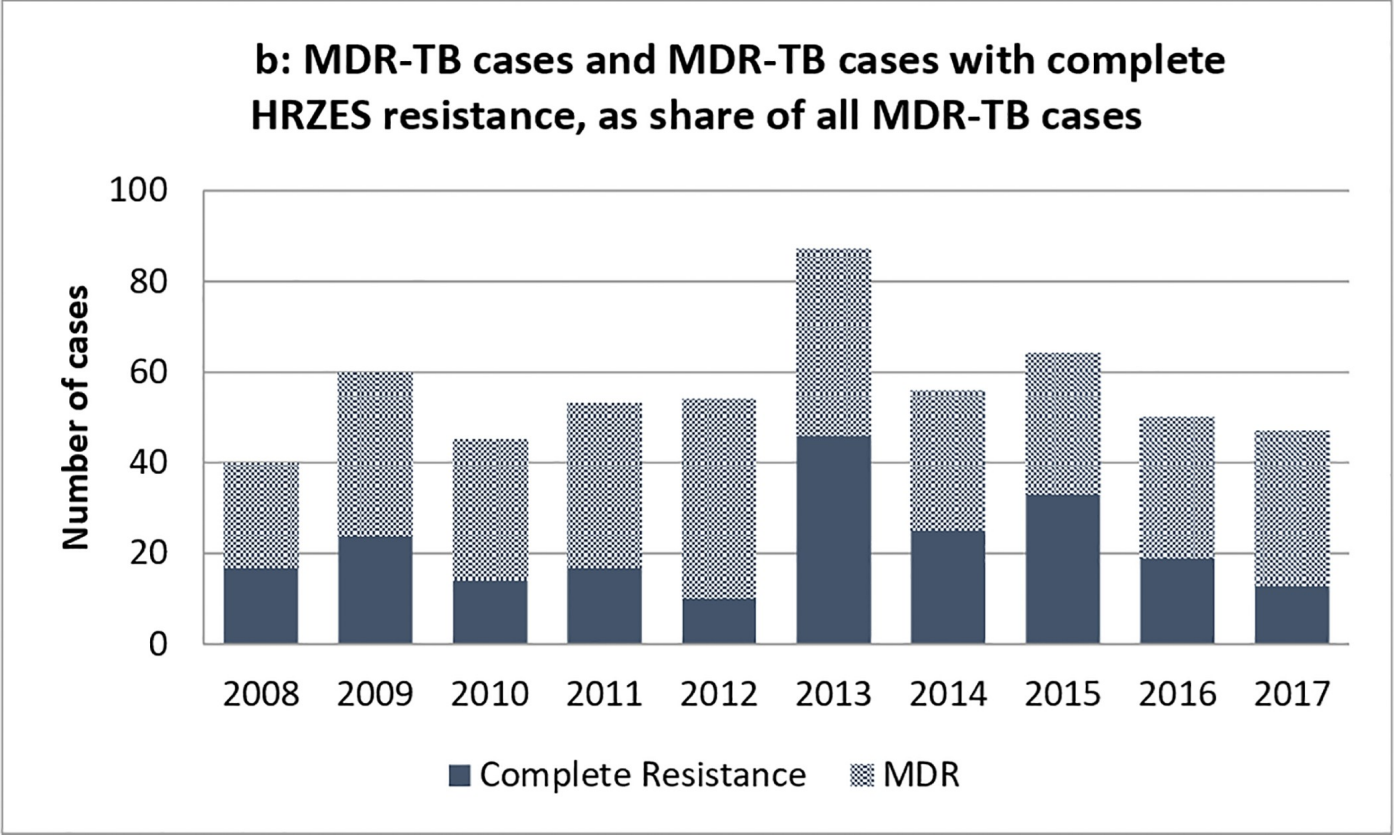
Rate of pan-resistance to first-line drugs among MDR-TB notifications.

The pooled national data revealed an overall proportion of any drug resistance of 3,324/26,228 (12.7%) in Germany (Fig 1). No significant overall changes could be observed for H, R, Z, and E monoresistance rates between 2008 and 2017 (p>0.01 for all; Fig 2), while S-monoresistance rates showed a significantly increasing trend over time (p<0.01, respectively) (Fig 2). However, the increasing trend was present until 2016 and the S-monoresistance rates declined in 2017. Overall, MDR-TB was found in 2.1% of cases in Germany but showed a significantly increasing trend over the study period (p<0.01; Figs 2 and 3). MDR-TB accounted for 40/2,891 (1.4%) TB cases in 2008 and for 47/1830 (2.6%) in 2017. Among MDR-TB cases, no clear trend was observed in the rates of resistance to all five drugs, but a remarkable increase from 2012 (10/54 (18.5%) of MDR-TB cases) to 2013 (46/87, 52.9% of MDR-TB cases) (Fig 3).

### Resistance patterns overall

Among the 26,228 cases with complete DST results, 3,324 (12.7%) were resistant to at least one of the five drugs (any resistance) (Fig 1). Out of 31 possible resistance patterns, 28 could be identified in the surveillance data, of which 4 patterns accounted for more than ¾ of all drug resistant cases (for S: 24.5%; H: 23.1%, HS: 16.6%; Z: 12.4%; Fig 1). Three possible patterns were not represented (RZE, RZS and ZES) and one pattern was only found once (HZE). Overall, 556 (2.1%) cases had MDR-TB, of which 218/556 (39.2%) showed resistance against all five drugs, 143/556 (25.7%) were resistant to four drugs, 141/556 (25.4%) to three drugs and 54/556 (9.7%) were resistant to only H and R (Fig 1).

Any resistance to R was present in 643/3,324 (19.3%) cases and 556 (86.5%) of them had MDR-TB, i.e. additional resistance to at least H. 87 R resistant cases had no accompanying H resistance (Table 2), accounting for 0.3% of all included cases and 13.5% of all cases with any R resistance.

**Table 2:** TB drug resistance patterns for HRZES and their distribution among TB cases in Germany, 2008–2017 (N = 3,324). Please note that MDR-TB patterns are in grey.

H	R	Z	E	S	Number of cases	Percentage of cases
				x	816	24.6%
x					769	23.1%
x				x	551	16.6%
		x			412	12.4%
x	x	x	x	x	218	6.6%
x	x			x	112	3.4%
	x				70	2.1%
x	x	x		x	67	2.0%
x	x		x	x	65	2.0%
x	x				54	1.6%
x			x	x	36	1.1%
x		x			27	0.8%
x		x		x	24	0.7%
x	x	x			19	0.6%
			x		13	0.4%
x		x	x	x	12	0.4%
x	x	x	x		11	0.3%
x	x		x		10	0.3%
x			x		10	0.3%
	x	x			5	0.2%
	x			x	5	0.2%
			x	x	5	0.2%
	x		x		3	0.1%
		x		x	3	0.1%
	x	x	x	x	2	0.1%
	x		x	x	2	0.1%
		x	x		2	0.1%
x		x	x		1	0.0%
	x	x	x		0	0.0%
	x	x		x	0	0.0%
		x	x	x	0	0.0%

### Drug resistance according to sex

Within the study population, 9,627/26,228 (36.8%) cases were female and 16,556/26,228 (63.2%) were male (Table 1). 2,033/16,556 (12.3%) males and 1,280/9,627 (13.3%) females diagnosed with TB had any resistance. No significant difference between sexes could be observed with regard to any resistance, H monoresistance, combined resistance to HS and MDR-TB (p>0.01 for all, data not shown). However, females had a significantly higher proportion of Z-monoresistance (male: 222/2,033, 10.9%; female: 190/1,280, 14.8%, p<0.01), while males had a significantly higher proportion of S-monoresistance (male: 534/2,033, 26.7%; female: 279/1,280, 21.8%, p<0.01).

### Drug resistance according to country of birth

When examining the resistance patterns by country of birth, cases born in Russia, Somalia, Kazakhstan, Turkey, India, Vietnam, Romania, Eritrea, Pakistan and Afghanistan accounted for the highest numbers of drug resistant cases. Cases born in Russia, Kazakhstan and Vietnam presented the highest proportions of any drug resistance with 33.2%, 28.2%, and 26.7%, respectively. For MDR-TB, once again cases born in Russia (14.1%) and Kazakhstan (8.2%) presented the highest proportions, followed by Somalia (3.8%). Because of the recent shift in country of origin of migrants in Germany from predominantly Eastern European countries towards countries from the Horn of Africa and the Middle East, country rankings were also analyzed in two 5-year timespans (208–2012 and 2013–2017).Between 2008 and 2012, Turkey, Russia, and Kazakhstan, had the three highest absolute shares of resistances in Germany. However, between 2013 and 2017, African countries became more dominant and cases born in Somalia and Eritrea, having the top two absolute shares of resistances, with Romania ranked third. Table 3 shows the overall proportions of any drug resistance, any H-resistance, any R-resistance, and MDR-TB for the top 10 countries of birth from 2008 to 2017.

**Table 3:** Proportions of any drug resistance, any H resistance, any R resistance, and MDR-TB for the 10 countries of birth with highest notifications of drug resistant cases, ordered by total number of cases with any drug resistance (2008–2017).

	Number of cases	Any drug resistance		Any H resistance		Any R resistance		MDR-TB	
		N	%	N	%	N	%	N	%
Germany	10,969	913	8.3%	435	4.0%	96	0.9%	70	0.6%
Russia	810	269	33.2%	224	27.7%	120	14.8%	114	14.1%
Somalia	916	165	18.0%	109	11.9%	42	4.6%	35	3.8%
Kazakhstan	562	162	28.8%	136	24.2%	46	8.2%	46	8.2%
Turkey	1,269	148	11.7%	91	7.2%	6	0.5%	5	0.4%
India	816	109	13.4%	71	8.7%	20	2.5%	19	2.3%
Vietnam	389	104	26.7%	60	15.4%	9	2.3%	7	1.8%
Romania	899	103	11.5%	69	7.7%	36	4.0%	26	2.9%
Eritrea	668	98	14.7%	55	8.2%	12	1.8%	10	1.5%
Pakistan	530	94	17.7%	44	8.3%	9	1.7%	8	1.5%
Afghanistan	507	70	13.8%	40	7.9%	7	1.4%	5	1.0%
Total	18,335	2,235	12.2%	1,334	7.3%	403	2.2%	345	1.9%

### Resistance patterns according to age and country of birth

Overall, German-born cases had a significantly lower proportion of any resistance than foreign-born patients (German-born: 913/10,969, 8.3%, Foreign-born: 2,337/14,600 16.0%, p<0.01). In German-born cases, drug resistance was mainly attributable to monoresistances, especially H (228/913, 25.0%) and S (236/913, 25.8%; Table 4). Moreover, 96 German-born cases had any resistance to R (0.9%), among which 70 (72.9%) had MDR-TB and 20 (20.8%) had R monoresistance. In foreign-born cases, drug resistance was attributable to high proportions of MDR-TB (478/2,337, 20.5%), H (521/2,337, 22.3%) and S (561/2,337, 24.0%) monoresistances. 536 (3.7%) foreign-born cases had any R-resistance of which 478 (89.2%) cases had MDR-TB and 48 (9.0%) cases had R monoresistance. Foreign-born cases also had higher proportions of combined resistance against HS (foreign-born: 429/2,337, 18.4%, German-born: 109/913. 11.9%; p<0.01), especially in the age groups 40–59 years (Foreign-born: 150/633, 23.7%; German-born: 45/287, 15.7%; p<0.01) and 60+ years, with a notable difference in those aged 60+ (German-born: 28/395, 4.6%; foreign-born: 56/283, 19.8%; p<0.01). However, cases born in Germany had a higher proportion of Z-monoresistance (German-born: 206/913, 22.6%; foreign-born:197/2,337 8.4%, p<0.01), especially among those aged 60+ (German-born: 145/395, 36.7%; Foreign-born: 44/283, 15.5%, p<0.01).

**Table 4:** The 4 predominant patterns in drug-resistant culture positive TB cases with DST results, by country of birth and age group (for cases with available information), Germany 2008–2017, Isoniazid (H), Rifampicin (R), Pyrazinamide (Z), Ethambutol (E), and Streptomycin (S).

**Age group: < 15 yr.**						
Country of birth	Total No. of TB cases	Any resistance present	Among any resistance			
			H	Z	S	HS
Germany	366	64 (17.5%)	16 (25.0%)	6 (9.4%)	20 (31.3%)	12 (18.8%)
Other	209	39 (18.7%)	9 (23.1%)	6 (15.4%)	7 (17.9%)	5 (12.8%)
Total	575	103 (17.9%)	25 (24.3%)	12 (11.7%)	27 (26.2%)	17 (16.5%)
**Age group: 15–39 yr.**						
	Total No. of TB cases	Any resistance present	Among any resistance			
			H	Z	S	HS
Germany	1,979	167 (8.4%)	50 (29.9%)	10 (6.0%)	54 (32.3%)	24 (14.4%)
Other	8,323	1,381 (16.6%)	302 (21.9%)	101 (7.3%)	354 (25.6%)	218 (15.8%)
Total	10,302	1,548 (15.0%)	352 (22.7%)	111 (7.2%)	408 (26.4%)	242 (15.6%)
**Age group: 40–59 yr.**						
	Total No. of TB cases	Any resistance present	Among any resistance			
			H	Z	S	HS
Germany	3,449	287 (8.3%)	71 (24.7%)	45 (15.7%)	76 (26.5%)	45 (15.7%)
Other	3,542	633 (17.9%)	132 (20.9%)	46 (7.3%)	139 (22.0%)	150 (23.7%)
Total	6,991	920 (13.2%)	203 (22.1%)	91 (9.9%)	215 (23.4%)	195 (21.2%)
**Age group: 60+ yr.**						
	Total No. of TB cases	Any resistance present	Among any resistance			
			H	Z	S	HS
Germany	5,174	395 (7.6%)	91 (23.0%)	145 (36.7%)	86 (21.8%)	18 (4.6%)
Other	2,525	283 (11.2%)	78 (27.6%)	44 (15.5%)	61 (21.6%)	56 (19.8%)
Total	7,699	678 (8.8%)	169 (24.9%)	189 (27.9%)	147 (21.7%)	74 (10.9%)
**All age groups**						
	Total No. of TB cases	Any resistance present	Among any resistance			
			H	Z	S	HS
Germany	10,969	913 (8.3%)	228 (24.9%)	206 (22.6%)	236 (25.8%)	99 (10.8%)
Other	14,600	2,336 (16.0%)	521 (22.3%)	197 (8.4%)	561 (24.0%)	429 (18.4%)
Total	25,569	3,249 (12.7%)	749 (23.1%)	403 (12.4%)	797 (24.5%)	528 (16.3%)

Both groups showed a decreasing MDR-TB trend with increasing age (p<0.01 for both). However, cases born outside Germany were more affected by MDR-TB with 478/14,600 (3.3%) compared to 70/10,969 (0.6%) in German-born cases (p<0.01). For cases with MDR-TB, regardless of their origin, resistance against all 5 drugs was the most common pattern, accounting for 23/70 (32.9%) of MDR-TB cases born in Germany and 191/478 (40.0%) of cases born outside Germany (Table 5). Resistance against only H and R accounted for 10/70 (14.3%) of all MDR-TB cases among German-born cases and for 44/478 (9.2%) of cases born outside Germany.

**Table 5:** Multidrug resistance patterns in drug-resistant culture positive TB cases with DST results, by country of birth and age group (for cases with available information), Germany 2008–2017, Isoniazid/H, Rifampicin/R, Pyrazinamide/Z, Ethambutol/E, and Streptomycin/S.

**Age group: < 15 yr.**							
Country of birth	Total No. of TB cases	MDR-TB present		HR only	3 drugs	4 drugs	All 5 drugs
		N	%	N	N	N	N
Germany	366	6	1.6%	0	2	2	2
Other	209	8	3.8%	1	1	2	4
Total	575	14	2.4%	1	3	4	6
**Age group: 15–39 yr.**							
	Total No. of TB cases	MDR-TB present		HR only	3 drugs	4 drugs	All 5 drugs
		N	%	N	N	N	N
Germany	1,979	19	1.0%	2	6	3	8
Other	8,323	318	3.8%	27	71	79	141
Total	10,302	337	3.3%	29	77	82	149
**Age group: 40–59 yr.**							
	Total No. of TB cases	MDR-TB present		HR only	3 drugs	4 drugs	All 5 drugs
		N	%	N	N	N	N
Germany	3,449	29	0.8%	5	10	7	7
Other	3,542	122	3.4%	11	36	33	42
Total	6,991	151	2.2%	16	46	40	49
**Age group: 60+ yr.**							
	Total No. of TB cases	MDR-TB present		HR only	3 drugs	4 drugs	All 5 drugs
		N	%	N	N	N	N
Germany	5,174	16	0.3%	3	1	6	6
Other	2,525	30	1.2%	5	12	9	4
Total	7,699	46	0.6%	8	13	15	10
**All age groups**							
	Total No. of TB cases	MDR-TB present		HR only	3 drugs	4 drugs	All 5 drugs
		N	%	N	N	N	N
Germany	10,969	70	0.6%	10	19	18	23
Other	14,600	478	3.3%	44	120	123	191
Total	25,569	548	2.1%	54	139	141	214

### Drug resistance according to prior TB

Overall, 2,820/22,555 (12.5%) of all cases reported having prior TB diagnosis, while the majority (19,735/22,555, 87.5%) reported not having a prior disease. Cases with prior TB had a significantly higher proportion of any resistance, with 516/2,820 (18.3%) in comparison to 2.303/19,735 (11.7%) among new cases (p<0.01). While new cases presented significantly higher proportions of S (new cases: 614/2,303, 26.7%; previous cases: 81/516, 15.7%; p<0.01) and Z monoresistance (new cases: 300/2,303, 13.0%; previous cases: 43/516, 8.3%; p<0.01), previous cases had a higher MDR-TB proportion with 186/2,820 (6.6%) in comparison to 281/19,735(1.4%, p<0.01) for new cases. Among MDR-TB cases, resistance against all five drugs (HRZES) was once again the predominant pattern for both groups, accounting for 92/281 (32.7%) of new and 89/186 (47.8%) of previous MDR-TB cases. After further stratification for country of birth, only foreign-born cases still showed significant differences in the proportions of any resistance and MDR-TB between new and previous cases. Specifically, 1,553/10,630 (14.6%) of foreign-born new cases and 367/1,352 (27.1%) of previous cases had any resistance and 224/10,630 (2.1%) of foreign-born new cases and 171/1,352 (12.6%) of previous cases had MDR-TB.

### Drug resistance according to site of disease

Within the study population 21,254/26,195 cases (81.1%) had pulmonary TB and 4,941/26,195 (18.9%) cases had extrapulmonary TB. For any resistance, there was no significant difference between pulmonary TB cases (2,646/21,254, 13.6%) and extrapulmonary TB cases (671/4,941, 12.4%, p>0.01). For monoresistances, however, cases with pulmonary TB had a significantly lower proportion of Z monoresistance (pulmonary: 263/2,646, 9.9%; extrapulmonary: 148/671, 22.1%, p<0.01). In contrast, cases with pulmonary TB had a higher occurrence of MDR-TB with 493/21,254 (2.3%) in comparison to 62/4,941 (1.3%) of cases with extrapulmonary TB (p<0.01). Full resistance against HRZES was once again predominant among MDR-TB cases, being reported in 196/496 (39.8%) of pulmonary MDR-TB cases and 22/62 (35.5%) of extrapulmonary MDR-TB cases.

### Regression analyses

The final multivariable logistic regression results are presented in Table 6. Compared to adults, children had higher odds of having any drug resistance (OR = 1.59; 95% CI: 1.27, 1.97), MDR-TB (OR = 1.74; 95% CI: 1.03, 2.77), and resistance against all five drugs (OR = 2.08; 95% CI: 1.24, 4.42). In contrast, elderly had lower odds for all three resistance patterns compared to adults. Males had significantly lower odds of having any resistance in comparison to female cases (OR = 0.90; 95% CI: 0.84,0.98), but no significant effect of sex could be found for MDR-TB and resistance against all five drugs. Cases born outside of Germany had almost twice the odds of having any resistance versus cases born in Germany (OR = 2.04; 95% CI: 1.87,2.22). This increased to almost 4.5 times higher odds for having MDR-TB (OR = 4.4.71; 95% CI: 3.70,6.10) and a more than 3 times higher odds for having resistance against all five drugs (OR = 3.86; 95% CI: 2.51,6.22). Cases with prior TB diagnosis had approximately double the odds of having any resistance versus those with no prior TB diagnosis (OR = 1.95; 95% CI: 1.76,2.16); this increased to almost 6 times higher odds for MDR-TB (OR = 5.95; 95% CI: 5.00,7.06) and 9 times higher odds for resistance against all five drugs (OR = 9.01; 95% CI: 6.66,12.19). Lastly, cases with pulmonary TB had twice the odds of having both MDR-TB (OR = 2.00; 95% CI = 1.57,2.61) and resistance against all five drugs (OR = 2.12; 95% CI: 1.33,3.60) versus cases with extrapulmonary TB.

**Table 6:** Results of multivariable logistic regression analysis of factors associated with any drug resistance (HRESZ), multidrug resistant TB, and resistance against all five drugs (HRESZ), 2008–2017.

Risk factor	Any drug resistance			Multidrug resistance (MDR-TB)			Resistance against all five drugs (HRZES)		
	OR	95% CI		OR	95% CI		OR	95% CI	
		lower	upper		lower	upper		lower	upper
**Age (years)**									
Adult (<15)	ref			ref			ref		
Child (15–59)	1.59	1.27	1.97	1.74	1.03	2.77	2.02	0.78	4.30
Elderly (60+)	0.64	0.58	0.70	0.24	0.18	0.32	0.12	0.05	0.23
**Sex**									
Female	ref			ref			ref		
Male	0.90	0.84	0.98	0.95	0.80	1.13	0.81	0.60	1.11
**Country of birth**									
Germany	ref			ref			ref		
Not Germany	2.04	1.87	2.22	4.71	3.70	6.10	3.85	2.50	6.20
**Prior TB**									
No	ref			ref			ref		
Yes	1.95	1.76	2.16	5.95	5.00	7.06	9.06	6.66	12.26
**Main site of TB**									
Extrapulmonary	ref			ref			ref		
Pulmonary	0.98	0.89	1.07	2.00	1.57	2.61	2.17	1.36	3.69

## Discussion

Our study is one of the limited number of recent studies worldwide to analyze national surveillance data on drug resistance patterns over a long period of time. Our analysis revealed an overall any drug resistance proportion of 12.7% in Germany, among which four resistance patterns were clearly predominant and accounted for more than ¾ of all drug resistant cases in–monoresistance against S, monoresistance against H, combined resistance against HS and monoresistance against Z. Monoresistance against S was the most prevalent drug resistance pattern in cases born in Germany and outside Germany, as well as among all age groups, very likely due to the long historical usage. These findings confirm that the withdrawal of S as a first-line drug in 2012 in Germany [38] has minimal relevance for TB therapy due to high resistance levels against it in the population. As a result, it can be questioned whether systematic surveillance of S resistance is still essential.

Resistance against H presents another highly prominent drug resistance pattern among tuberculosis patients in Germany. Our analysis showed high proportions of H resistance among all subpopulations, but cases originating from Eastern Europe showed remarkably high proportions of resistance. This supports the use of either 3–4 months of combined H and R therapy or 4 months of R monotherapy for LTBI treatment in Germany [19].

We also found unexpectedly high proportions of S and H resistances among children. A potential explanation for this finding might be the presence of selection bias as bacteriological confirmation is challenging in paucibacillary childhood TB and diagnosis may, as a result, be intensified particularly in children with unknown infection sources and suspected drug resistance.

Z monoresistance was another significant finding in our analysis. We saw a remarkably high proportion of Z resistance among the German-born elderly population (60+), being almost 2.5-times higher than the proportion in the foreign-born elderly. One hypothesis might be that elderly cases present higher proportions of prior, agriculturally-acquired infection with *Mycobacterium bovis*, which is inherently resistant to Z. In fact, of the 145 German-born elderly cases with monoresistance to Z, 95 (65.5%) were reported as having *M*. *bovis* as the species of infection. Apart from that, Z-monoresistance was 2.2-times higher in extrapulmonary cases in comparison to pulmonary cases. Some studies have previously indicated that infection with *M*. *bovis* more often leads to extrapulmonary TB disease [40, 41]. As a result, species specification of TB infection is an important step to support appropriate design of treatment regimens.

Between 2008 and 2017, 2.1% of all cases in Germany had MDR-TB. In comparison to the European average of 3.7% in 2016, Germany presents a low proportion of MDR-TB cases [7]. Nevertheless, in contrast to the European-wide trend [7], we observed a slight increase in MDR-TB cases notified over the last 10 years, the reasons for which are likely the increase in migration from high MDR-burden regions, but perhaps also better reporting by local health authorities. Especially of note is that we rarely observed “simple” MDR-TB, i.e. resistance to only H and R and MDR-TB cases mostly presented additional drug resistances. Approximately 40% of MDR-TB cases in our study were resistant to all 5 drugs (HRZES) and only 10% were resistant to H and R only. This supports the WHO recommendation to initiate MDR-TB treatment with four second-line anti-TB drugs and not to rely on any first-line drugs, until detailed DST results are available [18].

Cases with prior TB disease and foreign-born cases have a high risk of MDR-TB. Our analysis showed that cases with prior TB diagnosis had an almost six times higher odds of having MDR-TB and nine times higher odds of having resistance against all five drugs in comparison to new cases. A number of studies have analyzed risk factors associated with MDR-TB [42–53] for different populations around the world. All of them found prior treatment to be one of the most significant and consistent predictors for the presence of drug resistance. In our study, although we utilized prior TB diagnosis as a proxy for prior TB treatment, our results are consistent with these previous results. Nevertheless, MDR-TB, and especially resistance against all five drugs, was also prevalent in new cases and in children, confirming that MDR-TB strains are endemic in many settings and that MDR-TB is no longer only a matter of acquired drug resistance.

Cases born outside Germany had a 5.5-fold higher proportion of MDR-TB in comparison to cases born in Germany. Several studies have also found higher proportions of MDR-TB among migrants in comparison to the local populations [44, 47, 48, 50, 51]. Cases from Eastern Europe, especially Russia and Kazakhstan, accounted for a substantial proportion of cases with MDR-TB in our study. This is in line with findings from Falzon and colleagues [50] showing that MDR-TB is strongly associated with origin from the former Soviet countries. Surveillance figures further show that Eastern European countries account for 85% of the TB burden and 99% of the MDR-TB cases in the WHO European region [9, 50]. However, due to the arrival of a substantial number of refugees and asylum seekers into Germany from 2014–2016, the migration pattern in Germany has undergone a changed. For example, African countries, among them countries with a high burden of drug resistant TB, such as Somalia and Eritrea, have recently become prominent in the drug resistance landscape, accounting for a considerable proportion of drug resistant cases. Between 2013 and 2017, cases from Somalia and Eritrea accounted for 11.2% and 7.2%, respectively, of all foreign-born cases with any resistance (2008–2013: 2.3% and 0.7%) and for 10.9% and 2.9% of all foreign-born MDR-TB cases (2008–2013: 1.0% for both). MDR-TB cases from countries in the Horn of Africa, such as Somalia and Eritrea, were also the subject of a large Europe-wide outbreak investigation in 2016, where the majority of the cases in the cluster were reported in Germany [54]. Nevertheless, the overall proportion of MDR TB remained stable at 2–3%, and we also found MDR-TB in cases born in Germany, especially among younger, German-born cases, which highlights the continued need for rapid DST and strengthened bacteriological confirmation among all age groups.

Rapid DST techniques are meaningful, especially in patients with high risk of drug-resistant TB, in order to rapidly commence adequate treatment before phenotypic DST results become available. Recently, R resistance has increasingly been considered as a surrogate marker for MDR-TB [23]. Although R monoresistance is rare worldwide [38], it seems to be prevalent in Germany. 13.5% of R-resistant cases in our study did not have H-resistance and among those without accompanying H-resistance, about 80% of all R-resistant cases were monoresistant.

Together with the high proportion of H resistance in Germany and our finding that MDR-TB is rarely only HR resistance in Germany, our results raise some concerns about the possibility of incorrect diagnosis of drug-resistant TB in Germany if currently available rapid tests were to be used as the only diagnostic tests. Focusing on only R resistance or HR resistance, other important and relevant resistance patterns would be overlooked unless additional testing is conducted [55]. Although more resource-intensive, comprehensive genotypic testing using whole genome sequencing has shown high concordance with conventional phenotypic techniques for first line TB drugs and could, therefore, be another relevant rapid test technology in resource-rich countries in the future [55].

In our multivariable regression analyses, we found that being born outside of Germany and prior TB disease diagnosis were the only consistent risk factors associated with having any resistance, MDR-TB, and complete first-line resistance. In line with our findings, most studies do not report any significant association between sex and MDR-TB [42, 43, 45, 56, 57]. Nevertheless, Faustini, Hall and Perucci [44] report male sex to be a risk factor, while Liu et. al. [52] found an association between female sex and MDR-TB. Several other studies have found younger age to be associated with the development of MDR-TB [44, 46, 49, 52, 53]. We could not support these findings with the results from our multivariable analysis, but results from our descriptive analysis indicate a similar pattern.

Our study has a number of limitations. Our study is based on reported surveillance data and we did not have insight into the original laboratory results or into detailed DST information on the cases. Because our data includes a considerable number of migrant cases, who can be a mobile population group, our surveillance data is incomplete for a number of variables, especially on DST. Of the 48,044 notified active TB cases between 2008 and 2017 in Germany, 76.8% had DST results available for HRZES. Although this is above the European average, notified DST testing rates in Germany do not meet ECDC’s target for TB elimination [7]. According to the ECDC, performing cultures and DST in at least 80% of the cases is a necessary step to achieve the elimination of TB in Europe [58].As a result, our lower DST testing coverage should be interpreted with caution. Moreover, DST for E and Z are less reliable than DST for H and R, especially in MDR-TB cases. Information on resistance against second-line drugs is limited and could therefore not be included in our analysis. Information on social determinants and behavioural risk factors such as HIV status, unemployment, alcohol and drug abuse, smoking, and diabetes is not reported at the national level of the the notification system and could also not be included in the risk factor analysis. Since data on prior treatment was incomplete, we utilized prior TB diagnosis as proxy, but this may mean that we may have missed relevant information on the type of prior treatment and its outcome.

## Conclusion

Drug resistance patterns observed in Germany, particularly for MDR-TB cases, are far more complex than expected. Although the overall proportions of drug-resistance and MDR-TB are low, most MDR-TB cases in Germany present with additional resistances against other standard anti-TB drugs and almost 40% of MDR cases showed complete resistance against all five standard TB drugs (HRZES). As drug resistance patterns vary significantly in different subgroups, our findings highlight the importance of considering demographic characteristics of cases and knowing the patients’ full drug resistance profile to tailor treatment for optimal outcome. This is especially significant as our study found higher than expected proportions of drug resistances and MDR-TB among children and German-born adults. Finally, our finding of considerably high H monoresistance rates is important towards informing LTBI treatment regimens in Germany.
